# Colonization efficiency of *Pseudomonas putida* is influenced by Fis-controlled transcription of *nuoA-N* operon

**DOI:** 10.1371/journal.pone.0201841

**Published:** 2018-08-02

**Authors:** Annika Teppo, Andrio Lahesaare, Hanna Ainelo, Kadri Samuel, Maia Kivisaar, Riho Teras

**Affiliations:** Chair of Genetics, Institute of Molecular and Cell Biology, University of Tartu, Tartu, Estonia; Academia Sinica, TAIWAN

## Abstract

Root colonization of plant growth-promoting bacteria is a complex multistep process that is influenced by several factors. For example, during adherence to plant roots, bacteria have to endure reactive oxygen species (ROS) produced by plants. In this study, we report that the global transcriptional regulator Fis is involved in the regulation of ROS-tolerance of *Pseudomonas putida* and thereby affects barley root colonization. *Fis* overexpression reduced both ROS-tolerance and adherence to barley roots and activated the transcription of the *nuoA-N* operon encoding NADH dehydrogenase I, the first enzyme of a membrane-bound electron-transport chain. The *nuoA-N* knockout mutation in the *fis*-overexpression background increased the ROS-tolerance and adherence to barley roots. We show that *nuoA* has two transcriptional start sites located 104 and 377 nucleotides upstream of the coding sequence, indicating the presence of two promoters. The DNase I footprint analysis revealed four Fis binding sites: Fis-nuo1 to Fis-nuo4, situated between these two promoters. Site-directed mutagenesis in a promoter-lacZ reporter and β-galactosidase assay further confirmed direct binding of Fis to Fis-nuo2 and probably to Fis-nuo4 but not to Fis-nuo1 and Fis-nuo3. Additionally, the results implied that Fis binding to Fis-nuo4 could affect transcription of the *nuoA-N* operon by modification of upstream DNA topology. Moreover, our transposon mutagenesis results indicated that Fis might be involved in the regulation of several alternative ROS detoxification processes utilizing NADH.

## Introduction

Plant-microbe communication is an important process for root colonization by plant growth promoting bacteria (PGPB) like *Pseudomonas putida*. Plant roots secrete several organic and inorganic compounds that may attract or repel bacteria. Amino acids, organic acids, and saccharides are readily metabolized by PGPBs and stimulate root colonization [1–5]. In addition to the organic compounds, plant roots secrete inorganic molecules, including reactive oxygen species (ROS) such as hydrogen peroxide (H_2_O_2_), superoxide radical ($O_{2}^{-}$) and hydroxyl radical (HO^−^) [6]. Plants use ROS for signalling, programmed cell death, stress responses and protection against pathogenic microorganisms [6–9]. Pathogens induce a biphasic ROS production in plants, consisting of a low extent first phase, followed by a much higher and stable accumulation during the second phase [10–12]. Similarly to pathogens, plant growth promoting bacteria have to endure the reactive oxygen species produced by plant roots in the first stage, but they do not evoke the second phase of ROS secretion [13,14].

The root colonization process of rhizospheric bacteria is complex. Bacteria, even rhizospheric PGPBs have to detoxify exogenous ROS produced by plant roots during the colonization process [13–15]. At the same time, movement and attachment to the plant roots require energy, and energy production by oxidative phosphorylation is known to produce ROS [16]. The oxidative phosphorylation is the most effective mechanism for energy production, especially using NADH dehydrogenase I (encoded by the *nuoA-N* operon) for the first component in an electron transfer system [17,18]. NADH dehydrogenase I transports electrons from NADH to quinones and is an important complex for the generation of proton motive force [18,19]. This complex has been shown to produce ROS during electron transport from NADH to ubiquinone [16]. However, the deletion of *nuoA-N* operon from *E*. *coli* did not reduce the amount of endogenous ROS in the cells, despite that, the lack of fumarate-reducing flavoenzymes NadB and Frd decreased the amount of H_2_O_2_ approximately 25% compared to the wild-type cells [20]. The authors suspected that amount of ROS in the *nuoA-N* deletion strain could be recovered by some other source of ROS [20]. Alternatively, increased endogenous ROS activates the expression of NADH-dependent peroxidases in *P*. *putida* and *P*. *aeruginosa* and thereby demand of cells for NADH [21]. Thus, the decline in the concentration of NADH may reduce ROS-tolerance.

Despite the possibility that this protein complex can produce ROS during electron transport, it is still needed for root colonization [15,16]. For example, this protein complex has been shown to be involved in *P*. *fluorescence* WCS365 tomato root tip colonization in gnotobiotic systems via enhancing bacterial competitiveness for nutrients [15]. The absence of NADH dehydrogenase I complex reduces the colonization efficiency of tomato rhizosphere in the presence of a competitor strain, and this effect is not complemented by NADH dehydrogenase II (*ndh*) [15].

We have previously shown that *fis*-overexpression influences root colonization by *P*. *putida* at a very early stage when bacteria adhere to the plant roots (Jakovleva et al., 2012). Additionally, we have shown that the amount of LapA, a key adhesin for bacterial adherence and biofilm formation, is up-regulated in the *fis*-overexpression strain of *P*. *putida* [22,23]. Thus, the *fis*-overexpression should facilitate rather than reduce the adherence of *P*. *putida* to plant roots. Therefore, our goal was to determine whether Fis could be involved in the ROS metabolism in *P*. *putida* and also, whether the decrease of the root colonization ability of the *fis*-overexpression strain might be the result of the increased sensitivity to ROS produced by plant roots. Therefore we were primarily interested in investigating of the adherence of *P*. *putida* to the barley roots when ROS-production was up-regulated by gallic acid.

Fis is a nucleoid-associated protein that is well-studied in enterobacteria. It participates in several processes like transcriptional regulation of numerous genes, recombination, and replication [24,25]. Contrary to enterobacteria, Fis is considered an essential protein in pseudomonads and all our attempts to downregulate the expression of *fis* have been unsuccessful [24,26]. Because of this, we can only examine the impact of Fis on *P*. *putida* using the *fis*-overexpression strain F15.

In this study, we show that the overexpression of *fis* in *P*. *putida* causes enhanced sensitivity to ROS. We carried out transposon mutagenesis to find possible Fis regulated genes, which expression could affect the tolerance of *P*. *putida* to ROS on barley roots. As most hits were in the *nuoA-N* operon genes, we assessed the influence of Fis on the transcription of the *nuoA* gene and the effect of the *nuo* genes on the adherence to barley roots. We ascertained that Fis binds directly to the *nuo* promoter and activates the transcription of the *nuoA* gene. The increased expression of the *nuo* genes affects ROS-tolerance and can therefore decrease the barley root colonization of the *fis*-overexpressing *P*. *putida*.

## Experimental procedures

### Bacterial strains, plasmids, and media

The bacterial strains and plasmids used in this study are described in S1 Table. Bacteria were grown in complete LB medium (Miller, 1992). Solid LB medium contained 1.5% Difco agar. Antibiotics were added at the following concentrations: 100 μg ampicillin ml^−1^, 10 μg gentamicin ml^−1^, 50 μg kanamycin ml^−1^, 1.5 mg benzylpenicillin ml^−1^, 200 μg streptomycin ml^−1^. *E*. *coli* was incubated at 37°C and *P*. *putida* at 30°C. Bacteria were electrotransformed as described by Sharma and Schimke [27].

### DNA manipulations

For the construction of the *P*. *putida* F15 ΔnuoA-N, the DNA regions that flanked the Δ*nuoA-N* operon were cloned into the suicide vector pEMG using the protocol described by Martines-Garcia and de Lorenzo (2011). The 351-bp-long region located upstream of the *nuoA-N* operon was amplified by primers PP4119-fw, and nuo-1-rev (S2 Table), and the 534-bp-long region downstream of the *nuoA-N* operon was amplified by primers nuo-2-fw and nuo-2-rev (S2 Table). The amplified upstream and downstream regions of the *nuoA-N* operon were joined together by overlap extension-PCR [28]. After that, the 885-bp PCR fragment was purified and cloned into pEMG using the BamHI site, resulting in pEMG-ΔnuoA-N (S1 Table).

To overexpress the catalase gene *katA* in the *fis-*overexpression strain F15, we expressed this gene on a p9TTB, a derivate of low-copy-number plasmid pPR9TT [29]. The P_*tac*_-*katA* transcriptional fusion was cut from the pKStackatA [30] with the restriction endonucleases HindIII and NotI. This fragment was inserted into the vector p9TTB opened with the same enzymes, yielding a plasmid p9_katA (S1 Table). The plasmid p9_katA was introduced into the F15 strain by electroporation.

The promoter-probe vector p9TTBlacZ, which has low basal activity [22,29], was used for a β-galactosidase assay and the *nuoA* upstream regions were cloned in front of the promoterless *lacZ* gene. The constructs for the verification of potential promoters were cloned as follows. The 249-bp-long *nuoA* promoter region carrying a putative promoter P_*N-I*_ and putative Fis binding sites Fis-nuo1 to Fis-nuo4 was amplified from the chromosomal DNA of *P*. *putida* PaW85 by the use of oligonucleotides PP4119-fw and PP4119-4-rev (S2 Table). The 191-bp-long PCR product carrying a putative promoter P_*N-II*_ was amplified by nuo-1-fw and PP4119-3-rev (S2 Table). Promoter probe vectors p9_P_nuoA_1mut and p9_P_nuoA_2mut carrying four substitutions in potential -10 boxes were constructed similarly to previous constructs, but with one exception. Instead of the oligonucleotides PP4119-4-rev and PP4119-3-rev, the respective oligonucleotides PP4119-4-revmut and PP4119-3-revmut were used for amplification of *nuoA* promoter regions. The 567-bp-long PCR product carrying the putative promoters P_*N-I*_ and P_*N-II*_, and all the putative Fis binding sites was amplified by nuo-1-fw and PP4119-rev (S2 Table). The PCR-amplified DNA fragments were digested with BamHI and ligated into the BamHI-opened p9TTBlacZ in front of the reporter gene *lacZ*, resulting in plasmids p9_P_nuoA_1, p9_P_nuoA_1mut, p9_P_nuoA_2, p9_P_nuoA_2mut and p9_P_nuoA_12 (S1 Table).

Vectors for assessment of Fis’ influence to *nuoA* transcription were cloned by site-directed mutagenesis. The wild-type Fis-binding sites Fis-nuo1, Fis-nuo2, Fis-nuo3, Fis-nuo4 were mutated by two sequential PCRs, and the *P*. *putida* PSm chromosome was used as a template resulting in p9_P_nuoA_1-F1mut, p9_P_nuoA_1-F2mut, p9_P_nuoA_1-F3mut, and p9_P_nuoA_1-F4mut. These amplifications resulted in DNA fragments with five to seven substituted nucleotides in the Fis binding sites but otherwise identical to the DNA region present on the plasmid p9_P_nuoA_12. To substitute nucleotides in Fis-nuo1 and Fis-nuo2 binding sites, the oligonucleotides carrying substitutions (nuo1-mut and nuo2-mut) and PP4119-rev were used for the DNA amplification in the first PCR (S2 Table). In the second PCR, nuo-1-fw and the product of the first PCR were used as primers for DNA amplification from the *P*. *putida* PSm chromosome. To substitute nucleotides in the Fis-binding sites Fis-nuo3 and Fis-nuo4, the oligonucleotides carrying substitutions (nuo3-mut and nuo4-mut) and nuo-1-fw were used for DNA amplification in the first PCR (S2 Table). In the second PCR, PP4119-rev and the product of the first PCR were used as primers for DNA amplification from the *P*. *putida* PSm chromosome. The PCR fragments were after that cleaved with BamHI and cloned to p9TTBlacZ opened by BamHI, resulting in p9_P_nuoA_12-F1mut, p9_P_nuoA_12-F2mut, p9_P_nuoA_12-F3mut, and p9_P_nuoA_12-F4mut.

All designed constructs were verified by DNA sequencing to exclude PCR-generated errors in the cloned DNA fragments.

### Surface sterilization, seed germination, and root colonization assay

Barley seeds were first surface-sterilized in diluted Ace bleach (1:10) for 10 minutes, then in 75% ethanol for 1 minutes, and finally rinsed thoroughly with sterile distilled water [31]. Surface-sterilized seeds were germinated on wetted filter paper at room temperature. For the root colonization assay, the barley seedlings were pre-germinated for three to six days depending on the growth of the roots. The seedlings with 3–5 cm long roots were used for the inoculation with *P*. *putida* overnight cultures. The overnight grown bacteria were washed once with M9 buffer (Adams, 1959) and resuspended in the MS medium (Murashige & Skoog, 1962) to OD_580_ ~1. Germinated barley seeds were inoculated with the appropriate *P*. *putida* strains by submerging in 100 ml of bacterial suspension without shaking at room temperature for 30 minutes. Then the barley seedlings were washed once with M9 buffer to remove bacteria that did not adhere to roots. To recover the bacteria from the roots after 30 minutes of inoculation, the roots were cut and then ground in 1 ml of sterile M9 buffer. Serial dilutions from grounded root mixtures and initial inoculation mixtures were plated onto the LB agar amended with appropriate antibiotics. The attachment efficiency was calculated as a number of colony-forming units (c.f.u) per 0.1 grams of roots divided by the number of c.f.u from the initial inoculation mixture.

### Detecting ROS on the root surface and in the *P*. *putida* cell lysates

The barley seeds were surface-sterilized and germinated for 3 and 6 days as previously described. The roots were treated for 30 minutes with 0.05 mM, 0.1 mM or 1 mM gallic acid to see the stable induction of ROS in barley roots. The control seeds were not treated with gallic acid. The roots were removed from the seeds and incubated in 0.5 ml of sterile M9 buffer with occasional gentle shaking for 10 minutes. The M9 buffer without barley roots was used as a negative control and treated the same way as the M9 with the roots. ROS production was detected by measuring the fluorescence of oxidized dihydroethidium (DHE) as described previously [30]. The relative amount of ROS on the root surface was calculated per 0.1 g of barley roots as following:

$$X=\frac{(A-B)\times 0.1}{m}$$

where “A” was the fluorescence value of DHE in the reaction mixture, “B” was the fluorescence value of DHE in the M9 buffer and “m” the weight of barley roots in grams.

To measure the amount of endogenous ROS in *P*. *putida*, bacteria were grown in 5 ml of LB medium in the presence or absence of 1 mM IPTG for 18 hours. Approximately 2 × 10^9^ cells were collected by centrifugation and re-suspended in the M9 buffer. The cells were disrupted by sonication, and the cell lysate was divided into two equal amounts. The first half was used to measure the amount of endogenous ROS and the second half was cleared by centrifugation at 16,000 × *g* for 30 min at 4°C and used to determine the total amount of protein in a tryptophan assay [32]. The fluorescence of the reaction mixture without the cell lysates was used as a negative control. The relative amount of *P*. *putida* endogenous ROS was calculated as follows: the value of fluorescence of the reaction mixture was blanked against the fluorescence of the buffer. The value of the fluorescence was calculated per 1 mg of total proteins of *P*. *putida*. ROS production was detected by measuring the fluorescence of the product of dihydroethidium (DHE) as described previously [30].

### Estimation of the lethal concentration of H_2_O_2_ for *P*. *putida*

The strains were pre-grown overnight at 30°C in LB medium with or without 1 mM of IPTG supplementation. 1 ml of overnight grown bacteria were collected by centrifugation at 11,000 × *g* for 1 min at room temperature. Collected cells were washed with 1 ml of M9 buffer, collected by centrifugation and suspended in 1 ml of M9 buffer. 90 μl of cells were incubated for 30 min at room temperature in the presence of 16, 32, 64, 128, 256 and 512 mM of H_2_O_2_. After that, cells were collected by centrifugation at 11,000 × *g* for 3 min at room temperature and suspended in 90 μl of the M9 buffer. The control cells were treated similarly, except without H_2_O_2_ supplementation. The cell-suspensions were used for serial dilution spotting to assess viable cell counts. 5 μl from the mixture of the H_2_O_2_-tolerance assay were spotted on LB agar plates and incubated overnight at 30°C. The lethal concentration of H_2_O_2_ was determined as the concentration of H_2_O_2_ for which no colonies were detected on LB plate. Three biological replicas were performed and the representative results are shown.

### Detection of the *fis*’ expression in the strains of *P*. *putida*

Western immunoblot analysis was carried out to detect the amount of Fis from the crude lysates of *P*. *putida* grown in 50 ml LB broth supplemented with 0.5 mM, 1 mM IPTG or without IPTG supplementation. The cells were collected by centrifugation and sonicated in Fis purification buffer (100 mM Tris/HCl, pH 7.5, 0.3 M NaCl, 5% v/v glycerol). The cell lysates were centrifuged at 12 000 g for 30 min at 4°C. The total amount of protein in the cleared supernatant was measured spectrophotometrically by the content of tryptophan [32]. Proteins were separated by Tricine-SDS-PAGE (10%) electrophoresis [33], transferred to a membrane and the membrane was probed with mouse anti-Fis as previously described [23].

### Transposon mutagenesis and selection of F15 mutants with improved growth in the presence of ROS

For the identification of Fis-regulated genes that affect the sensitivity to ROS, the cells of F15 were subjected to mutagenesis by mini-Tn*5* containing the kanamycin resistance gene. The plasmid pBAM1 [34] was introduced by electroporation into *E*. *coli* strain DH5αλ*pir* [34]. The obtained donor-strain was mated with the helper plasmid-carrying strain *E*. *coli* HB101 and the *P*. *putida* recipient strain F15 [35]. The colonies tolerant to 300 μM of 4-nitroquinoline 1-oxide (NQO) were selected on LB medium plates supplemented with 1 mM IPTG. Four independent transposon mutagenesis experiments were carried out, resulting in estimated 40000 colonies that were tested. The presence of an intact *fis*-overexpression cassette in the chromosome was examined by PCR using the primers Prtac and fis-*Bam*HI (S2 Table). The second screening of the isolated transposon mutants was carried out in LB microtiter plates by cultivation in LB liquid medium supplemented with 1 mM IPTG, and 300 μM NQO and LB medium supplemented only with 1 mM IPTG. Bacteria were grown for 24 h at 30°C at 800 rpm, and the optical density of the cells was measured spectrophotometrically at 580 nm. The OD of the transposon mutants was compared to the OD of F15 by the following formula:

$$\left(\frac{{OD}_{T1}}{{OD}_{F15}}\times \frac{{OD}_{T2}}{{OD}_{F15}}\right)\times \left(\frac{{OD}_{T1}}{{OD}_{F15}}+\frac{{OD}_{T2}}{{OD}_{F15}}\right)$$

where OD_T1_ is the optical density of transposon mutants grown in the LB medium with 1 mM IPTG for 24 h. OD_T2_ is the optical density of transposon mutants grown in the LB medium with 1 mM IPTG and 300 μM NQO for 24 h, and OD_F15_ is the optical density of F15 grown in the LB medium with 1 mM IPTG and 300 μM NQO for 24 h. All transconjugants with a higher value than 1000 according to the presented formula were selected.

An arbitrary PCR of the genomic DNA was performed to localize mini-Tn*5* insertions in the chromosome of *P*. *putida*. The arbitrary PCR consists of two consecutive amplifications as described elsewhere [34,36]. A specific primer ME-I-uus complementary to mini-Tn*5* I-end and an arbitrary primer ARB6 were used [34] for the first amplification. The primers ME-I-uus2 and ARB2 were used for the second amplification [34]. The amplified fragments were sequenced to determine their genomic locations. The transposon mutants that had transposon insertions in the *fis*-overexpression cassette were excluded.

### Prediction of Fis-binding sites on promoter regions

Possible Fis-binding sequences on the promoter regions of the selected genes were predicted using the *E*. *coli* Fis-binding sites matrix [37] and the matrix-scan program available at the Regulatory Sequence Analysis Tools homepage (http://rsat.ulb.ac.be/). To predict potential Fis-binding sites *in silico* the -500 bp to +100 bp from the start-codon of the genes was studied. The Markov model of zero order (Bernoulli model), the organism-specific probability of nucleotides in the upstream region of genes in *P*. *putida* KT2440 and a P-value upper threshold of 1 × 10^−4^ were selected for the conditions of the background model. The rest of the parameters were left at the default values of the matrix-scan program.

### DNase I footprinting

DNase I footprint assays were performed for the identification Fis-binding sequences on the *nuoA* promoter region. PCR-amplified fragments were used for the DNase I footprint assay and were generated as follows. The 150 bp-long DNA fragment upstream of the *nuoA* gene was amplified using the nuoAup1 and nuoAdown2 oligonucleotides (S2 Table) to identify Fis binding sites Fis-nuo1 and Fis-nuo2 upstream of the *nuoA* gene. The 170 bp-long DNA fragment upstream of the *nuoA* gene was amplified using the PP4119-fw and nuoAdown1 oligonucleotides (S2 Table) to identify the Fis binding sites Fis-nuo3 and Fis-nuo4 upstream of the *nuoA* gene. Depending on the template, the PCR-amplified fragments contained either the wild-type or the mutated Fis-binding site. Labelling PCR products with [γ-^32^P]-ATP and preparing reaction mixtures and gel electrophoresis were carried out as described by Teras et al. [26].

### Gel mobility shift assay

The same labelled PCR-amplified fragments that were used for the DNase I footprint assays were also used for the gel mobility assay. Additionally, the non-labelled PCR product containing the Fis binding site LF2 [38] and a PCR product without the Fis-binding site RF1 [38] were used in out-competition experiments. The unlabelled DNA fragment LF2 was amplified using the oligonucleotides TnLsisse and SIDD-2 (S2 Table). Oligonucleotides PRH8 and Tnots (S2 Table) were used for the amplification of the unlabelled DNA RF1. The plasmids pLA1-12 and pRA1-12 [38] were used for amplifying LF2 and RF1, respectively. The amount of competing DNA in the reaction mixes was calculated in molecules.

Binding reactions with purified His-tagged Fis were carried out with 2 × 10^10^ molecules (750–1000 c.p.m.) of labelled DNA fragment in a reaction buffer (24 mM Tris/HCl pH 7.5, 50 mM KCl, 10 mM MgCl_2_, 1 mM CaCl_2_, 0.1 mM EDTA, 5% glycerol, 0.05 μg BSA μl^-1^ and 0.05 μg salmon sperm DNA μl^-1^) in a final volume of 20 μl. The mixtures were preincubated for 20 minutes at room temperature. After incubation, reaction mixtures were applied to a 5% non-denaturing polyacrylamide gel buffered with TBE (50 mM Tris, 60 mM boric acid, 5 mM EDTA; pH 7.5). Electrophoresis was carried out at 4°C at 10 V cm^-1^ for 3 h. The gels were vacuum-dried and exposed to a Typhoon Trio screen (GE Healthcare).

### Identification of 5´ ends of mRNA by RACE

The mRNA 5´ ends of the *nuoA* gene mRNA were identified by RACE (rapid amplification of cDNA ends) as described previously [39]. 1.5 μg of purified total RNA and the nuoA-RACE3 primer (S2 Table) were used for the amplification of the first strand of cDNA. The second strands of cDNA were amplified using the primer Adapt-pikkC (S2 Table), with 5´ ends binding according to poly-G, synthesized by terminal deoxynucleotidyltransferase (TdT) to the 3´ ends of the first strand of cDNA. For the second PCR, the primers Adapt-lyh and nuoA-RACE2 (S2 Table) were used. Zymo Research “The DNA Clean & Concentrator^TM^-5” kit was used for DNA purification between the RACE stages.

### Measurement of β-galactosidase activity

Β-Galactosidase activity was measured as described previously (Miller, 1992). *P*. *putida* cells were grown in the LB medium with or without 1 mM IPTG supplementation for 18 hours. As a source of β-galactosidase, the p9_P_nuoA_1, p9_P_nuoA_2, p9_P_nuoA_12, p9_P_nuoA_1-F1mut, p9_P_nuoA_1-F2mut, p9_P_nuoA_1-F3mut, p9_P_nuoA_1-F4mut constructs containing PP4119 promoter region in front of the *lacZ* reporter gene were used. At least eight independent measurements were performed.

### Statistical analysis

The multifactorial ANOVA and *post hoc* Bonferroni test at the significance level of 0.05 was used to assess the variability of experimental data. Data were checked for normality and, if necessary, transformed to common logarithm to obtain a normal distribution. The calculations were performed using Statistica 13 software.

## Results

### Overexpression of *fis* reduces the ROS-tolerance of *P*. *putida*

Plants use ROS for signalling, programmed cell death, stress responses and protection against pathogenic microorganisms [6–9]. As the *fis-*overexpressing *P*. *putida* strain F15 had reduced adherence to barley roots but at the same time formed stronger biofilm to abiotic surface compared to the wild-type cells [31], we hypothesized that *fis*-overexpression increases the sensitivity to ROS. In other words, *P*. *putida*’s viability decreases when *fis*-overexpressed cells are exposed to exogenous ROS.

**Fig 1:**
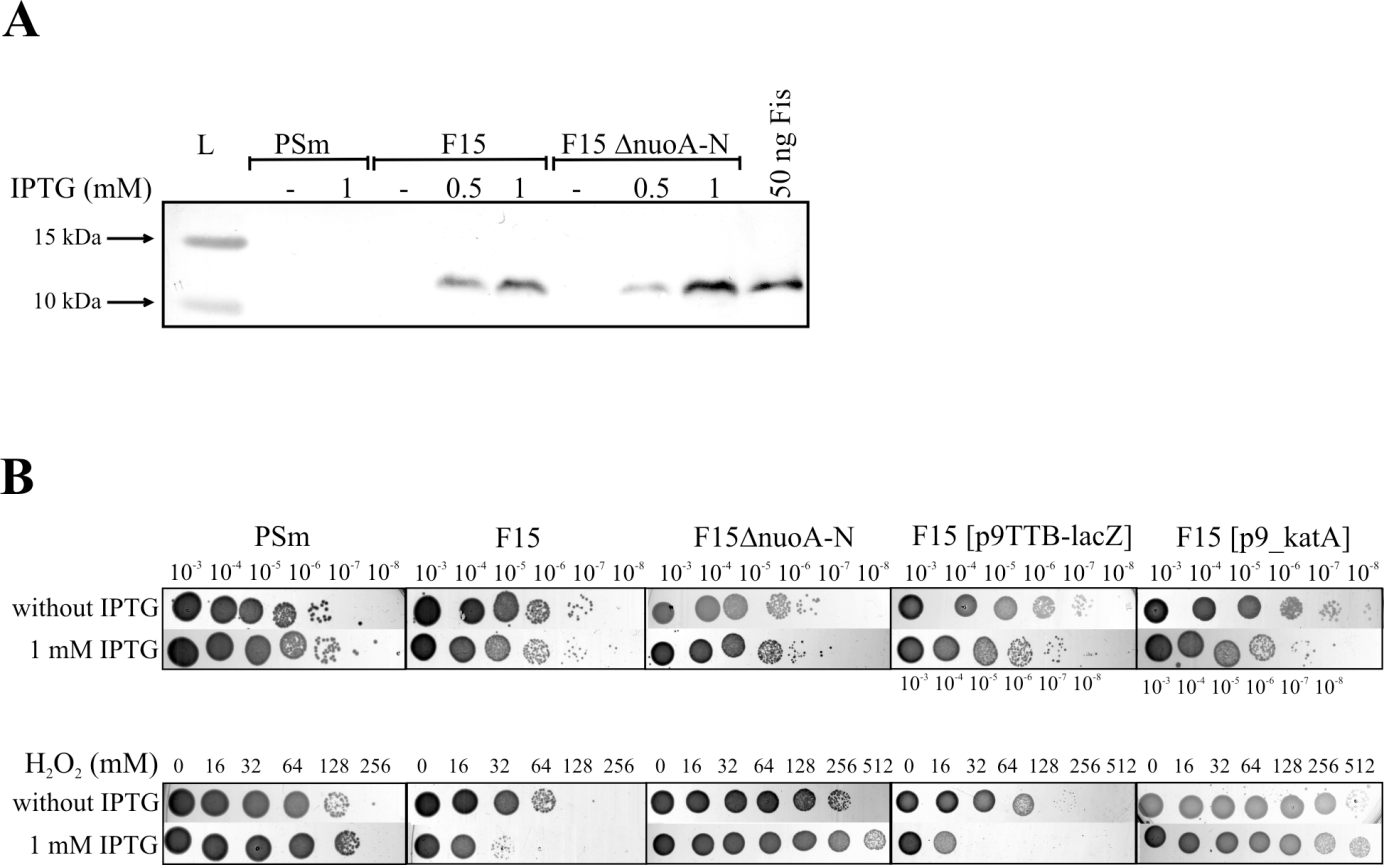
The *fis*-overexpression in *P*. *putida* by immunoblotting using polyclonal anti-Fis antibodies and comparison of H_2_O_2_-tolerance of *P*. *putida* strains. (A) The presence of Fis in thirty micrograms of crude cell lysates prepared from *P*. *putida* strains PSm, F15 and F15ΔnuoA-N grown in LB medium for 18 h were determined by immunoblot analysis. The supplementation of IPTG for *fis*-overexpression is shown above the lanes. Fifty nanograms of purified Fis (6His) were used as a positive control. Arrows show the location of marker proteins 15 and 10 kDa in size in the protein ladder (L) lane. (B) The strains PSm, F15, F15ΔnuoA-N and F15 harboring plasmids p9TTBlacZ as a negative control and p9-katA as a variant with an extra copy of *katA*. Overnight grown bacteria were washed and incubated in the M9 buffer in the presence of 0 to 512 mM H_2_O_2_ for 30 minutes followed by washing with M9 and spotting onto LB medium. The serial dilution spotting of *P*. *putida* without H_2_O_2_ is presented in the upper panel for assessing the c.f.u in the H_2_O_2_-tolerance assay. Spotted bacteria from H2O2-tolerance assay are shown in the bottom panel.

The overexpression of *fis* in *P*. *putida* strain F15 by immunoblot assay using polyclonal anti-Fis antibodies was verified (Fig 1A). F15 harbours an additional copy of the *fis* gene under the control of IPTG-inducible P_*tac*_ promoter [31]. As expected, the *fis* is overexpressed in F15 grown in LB medium supplemented with IPTG (Fig 1A). The H_2_O_2_-tolerance assay was used to assess the lethal dose of H_2_O_2_ for *P*. *putida* wild-type strain PSm and F15 using approximately same number of cells in reaction mixture (Fig 1B upper panel). The lethal concentration of H_2_O_2_ for *P*. *putida* wild-type strain PSm was 256 mM irrelevant to the presence or absence of IPTG in the pre-growth medium (Fig 1B bottom panel). Thus, the IPTG supplementation in the pre-growth medium of F15 will reveal the impact of *fis*-overexpression to the H_2_O_2_-tolerance. F15 pre-grown with 1 mM IPTG tolerated less H_2_O_2_ (lethal concentration of 64 mM) than F15 pre-grown without IPTG (lethal concentration of 128 mM, Fig 1B bottom panel). To prove that the loss of F15 viability was caused by H_2_O_2_, the extra copy of the catalase gene *katA* in the composition of p9_katA was introduced into the cells. The plasmid p9TTBlacZ was used as a negative control as peroxide and benzylpenicillin (resistance marker for the plasmid) can synergistically decrease cell viability. As expected, the overexpression of catalase in F15 efficiently protected bacteria against ROS irrespective of *fis*-overexpression (Fig 1B). According to these results, *fis-*overexpression reduced ROS-tolerance, and it was restored or even improved by the introduction of an extra *katA* copy.

### Adherence of *P*. *putida* to barley roots depends on the amount of ROS

**Fig 2:**
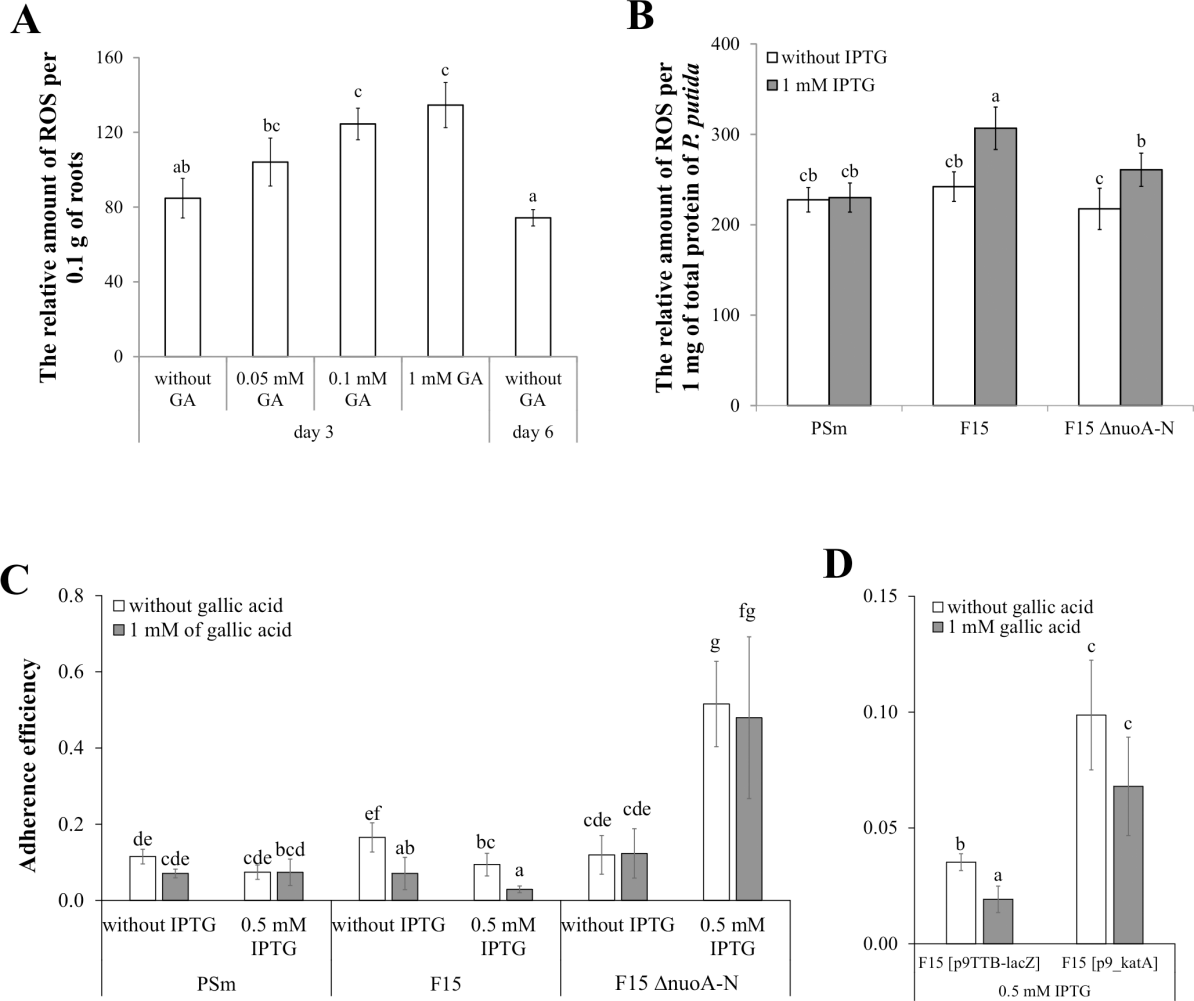
The relative amount of ROS produced on the surface of barley roots, in the cell lysates of *P*. *putida* and adherence efficiency of *P*. *putida* to barley roots. (A) The relative amount of ROS was defined as the fluorescence of the product of DHE blanked against the fluorescence of M9 buffer, which mean was 111.7 (SD 9), and thereafter calculated per 0.1 g barley roots. The roots were treated with 0.05 mM, 0.1 mM or 1 mM gallic acid for 30 minutes, or no gallic acid was added. Data from at least 8 independent measurements are shown. (B) The relative amount of ROS was defined as the fluorescence of the product of DHE calculated per 1 mg of total protein. Bacteria were grown in LB medium with or without 1 mM IPTG for 18 hours. Data from at least 4 biologically independent measurements are shown. *P*. *putida* strains were grown overnight in LB medium in the presence or absence of 0.5 mM IPTG, washed and applied to barley roots. Adherence efficiency was calculated as the ratio of c.f.u per 0.1 g of roots and c.f.u per 1 ml inoculation mixture. Data of at least 10 biologically independent measurements are shown. (C) Adherence efficiency of *P*. *putida* wild-type strain PSm, *fis*-overexpression strain F15 and F15ΔnuoA-N, the *nuoA-N* deletion variant from F15. (D) Adherence efficiency of *P*. *putida* F15 harboring an “empty” plasmid p9TTBlacZ and p9-katA containing an extra copy of *katA* gene. Data of at least 10 biologically independent measurements are shown. Error bars denote 95% confidence intervals of the means. Letters a-f depict statistical homogeneity groups according to ANOVA *post hoc* Bonferroni test. The same letters of the homogeneity groups denote non-significant differences (*P*>0.05) among the averages of the relative amount of ROS or adherence.

To determine whether the decreased tolerance of the *fis-*overexpression strain to ROS was the reason for the reduced adherence ability, we examined the adherence of *P*. *putida* to the barley roots when the level of ROS production was altered by the presence of gallic acid. Gallic acid is a phenolic compound that can generate elevated levels of ROS in the treated plant roots [40,41]. We examined whether it is possible to measure the level of ROS on barley roots with the dye dihydroethidium (DHE) and whether it is possible to use gallic acid to induce ROS production in barley roots (Fig 2). Indeed, DHE enabled to assess the relative amount of ROS on roots (Fig 2A). The amount of ROS was constant during the first days after germination, and the production of ROS was inducible by gallic acid (Fig 2A). Additionally, we assessed the possibility that the overexpression of *fis* increases the endogenous ROS in *P*. *putida*. Indeed, the relative amount of ROS was increased in cell lysates due to *fis* overexpression (Fig 2B).

After that, we assessed the effect of ROS on the adherence efficiency of *P*. *putida* to the barley roots, calculated as adhered bacteria on barley roots per bacteria in LB medium (Fig 2C). In this experiment the 0.5 mM of IPTG was used for overexpression of *fis* as previously published experiments of *P*. *putida*’s adhesion were done by using this concentration of IPTG. In general, the adherence efficiency of *P*. *putida* wild-type strain PSm did not depend on IPTG supplementation in growth medium or root treatment with gallic acid; and the adherence efficiency of wild-type and *fis*-overexpression strains were similar when barley roots without gallic acid treatment were used (Fig 2C). However, the assessment of IPTG supplementation to the adherence efficiency of F15 reveals the reduction of adherence when *fis* was overexpressed in *P*. *putida* by IPTG (Fig 2C), which concur with our previously published data [31]. Additionally, the gallic acid treatment of barley roots significantly reduced the ability of F15 to adhere to barley roots irrespective of the presence of IPTG in overnight LB medium (Fig 2C). In sum, it seems that wild-type strain of *P*. *putida* can cope with the negative effect of ROS in barley roots while the *fis*-overexpression strain can not. The artificial strain F15 may indicate the environmental factors, which are important in colonization process of *P*. *putida*. Therefore, understanding the ROS-sensitivity of *fis*-overexpression strain F15 can enlighten the colonization process of *P*. *putida*.

To investigate the involvement of ROS in the colonization process, we conducted barley root colonization experiments with the *fis-*overexpression strain F15 harbouring an additional catalase gene on plasmid p9_katA (Fig 2D). The same plasmid without the *katA* gene p9TTBlacZ was used for negative control. The overexpression of catalase in the strain F15 showed a positive effect on the adherence compared to the negative control p9TTBlacZ (Fig 2D). However, the presence of the plasmids in the *fis*-overexpression strain decreased the adherence efficiency of *P*. *putida*. Presumably, the presence of benzylpenicillin in the pre-growth medium had a negative impact on bacterial adherence to barley roots and therefore the adherence efficiency of F15 harbouring the control plasmid p9TTBlacZ was reduced.

These data indicated that *P*. *putida* wild-type strain PSm was insensitive to the enhanced amount of exogenous ROS on barley roots while *fis*-overexpression strain F15 was sensitive to it.

### The ROS-tolerance of *P*. *putida* F15 is alleviated by mini-Tn*5* insertions into the genes of the *nuoA-N* operon

To identify Fis-regulated genes that may affect ROS tolerance, we performed mini-Tn*5* transposon mutagenesis of the *fis*-overexpression strain F15 by a two-step selection in the presence of ROS-inducing 4-nitroquinoline 1-oxide (NQO). We focused on Fis-activated genes that reduced ROS-tolerance since we did not detect Fis-repressed genes by employing mini-Tn*5* that carried an outwardly orientated sigma70-type promoter (data not shown). The second stage of the screening was an evaluation of *fis*-overexpression’s random effect on the growth of transconjugants. The transconjugants with improved growth in the *fis*-overexpression background but still sensitive to ROS were eliminated at this stage. Eventually, 121 genes and 9 intergenic regions, which disruption with mini-Tn*5* increased the ability to grow in the presence of NQO, were identified (S3 Table) and 12 of them had two or more insertions in the same gene from different insertion events of mini-Tn*5* (S3 Table). For example, 18 transconjugants were selected with independent mini-Tn*5* insertion in the genes of the *nuoA-N* operon, encoding subunits of NADH dehydrogenase I (S3 Table). Six insertions were detected in the *nuoG* (PP_4124) gene, five in the *nuoCD* (PP_4121) and four in the *nuoJ* (PP_4127) gene (S3 Table). Three insertions were selected in the *lapA* (PP_0168) gene and in PP_2232 (S3 Table), encoding the adhesin LapA and an XRE family transcriptional regulator, respectively [42].

To confirm that those genes are involved in the ROS-tolerance, we studied the tolerance of most of the transconjugants on LB plates supplemented with H_2_O_2_ (S3 Table). 61 transposon mutants had increased tolerance to H_2_O_2_ compared to the Fis overexpression strain F15 (S3 Table). For example, the mini-Tn*5* insertion in the gene of NADH dehydrogenase I subunit *nuoCD* (PP_4121), methionine biosynthesis genes PP_5275 and *metR-1* (PP_1063) restored the H_2_O_2_-tolerance to wild-type level despite the *fis*-overexpression (S3 Table). The mini-Tn*5* insertions in the alpha-ketoglutarate metabolism genes *sucA* (PP_4189) and PP_4547 and also in the iron transport gene PP_0861 ensured increased tolerance to H_2_O_2_ compared to F15 (S3 Table).

Additionally, the putative Fis-binding sites on the upstream region of the selected genes or operons were predicted *in silico* (S3 Table). 22 genes had at least one potential Fis-binding site predicted *in silico*, indicating the possible regulation by Fis (S3 Table). The significant number of potential Fis-binding sites was predicted on the upstream regions of the *nuoA-N* operon (4 sites) and PP_0861 (3 sites, S3 Table).

As a significant number of transposon mutants had transposon insertions in the *nuo* operon genes, and four potential Fis-binding sites were predicted in the upstream sequence of the *nuoA* gene, we focused our subsequent research on the *nuoA-N* operon.

### Deletion of the *nuoA-N* operon alleviates the Fis-induced ROS-sensitivity in F15

To ascertain whether the improved ROS tolerance and enhanced barley root colonization are the results of the inactive *nuoA-N* operon in the *fis-*overexpression strain, the *nuoA-N* operon deletion strain F15ΔnuoA-N was constructed. Although we screened at least 1000 colonies, we were unable to delete the *nuoA-N* genes in the *P*. *putida* wild-type strain PSm (data not shown).

The *fis*-overexpression in F15ΔnuoA-N was controlled by immunoblot analysis using polyclonal anti-Fis antibodies (Fig 1A). The overexpression of *fis* in F15ΔnuoA-N by IPTG is similar to the F15 (Fig 1A).

The influence of the *fis*-overexpression to the ROS-tolerance of *P*. *putida* F15ΔnuoA-N was examined by exposing the cells to H_2_O_2_ for 30 minutes (Fig 1B). The deletion of the *nuoA-N* operon from F15 increased the viability of H_2_O_2_-exposed bacteria in the presence of IPTG compared to the cells grown without IPTG (Fig 1B). In sum, unlike F15, the ROS-tolerance of F15ΔnuoA-N did not decrease during *fis*-overexpression (Fig 1B).

The effect of *nuoA-N* deletion to the endogenous ROS during the *fis-*overexpression was moderate, hinting at an alternative mechanism to reduce the endogenous ROS in the cells. The elimination of the *nuo* genes decreased the amount of ROS in the lysates of the *fis-*overexpressing cells to a level comparable to the endogenous ROS of the wild-type strain PSm (P < 0.001; Fig 2B). The deletion of the *nuo* genes did not completely abolish the endogenous ROS enhancement by the Fis when overexpressed in the presence of IPTG, but it still decreased the overall level of ROS in the cells.

Additionally, the deletion of the *nuo* genes from the strain F15 also increased the adhesion of bacteria to the barley roots (Fig 2C). Unlike the original strain F15, the *fis-*overexpression by IPTG or the root treatment with gallic acid did not reduce the adherence of F15ΔnuoA-N (Fig 2C). Moreover, the presence of 0.5 mM IPTG in the pre-growth medium dramatically increased the adherence efficiency of F15ΔnuoA-N compared to the original strain F15 or to the wild-type strain PSm (Fig 2C). These results suggest that the *nuo* genes can affect ROS tolerance and thereby barley root colonization of the *fis*-overexpressing *P*. *putida*.

### Mapping the 5´ ends of *nuoA* mRNA and *nuoA* promoters

We observed that the overexpression of *fis* affects *P*. *putida* tolerance to ROS and colonization efficiency through the expression of the *nuo* genes; we were thus interested in determining whether Fis could regulate the transcription of the *nuoA* promoter directly.

**Fig 3:**
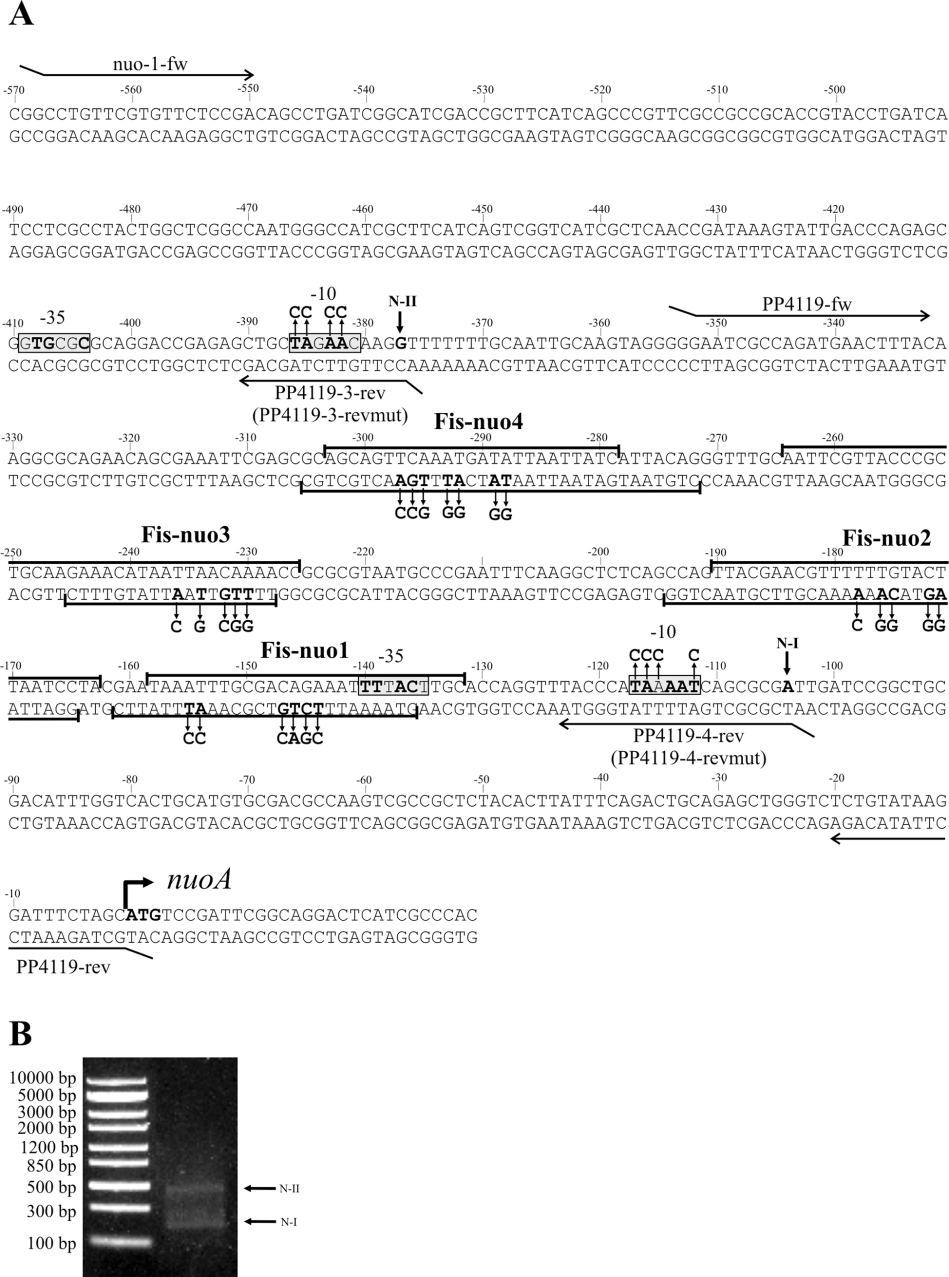
Mapped Fis binding sites, putative promoters, and 5´ends of mRNA at the *nuoA* promoter region. (A) The sequence of the *nuoA* promoter region. The ATG start codon of the *nuoA* gene is shown in bold. The first nucleotides of the mRNA 5´ ends are written in bold and designated as N-I and N-II. The potential –10 and –35 elements of the *nuoA* promoters (P_*N-I*_ and P_*N-II*_, respectively) are shown in grey boxes. The nucleotides in -10 and -35 box corresponding to *P*. *putida* σ^70^-type promoter consensus (TTGACC-N_17_-TATAC/aT) are indicated in bold. The Fis binding sites are shown in black brackets. The point mutations in the Fis-nuo1, Fis-nuo2, Fis-nuo3, Fis-nuo4 and -10 boxes of promoters are indicated by arrows. The oligonucleotides PP4119-fw and PP4119-4-rev were used for the construction of p9_P_nuoA_1, nuo-1-fw and PP4119-3-rev were used for the construction of p9_P_nuoA_2, and nuo-1-fw and PP4119-rev were used for the construction of p9_P_nuoA_12. (B) Agarose-gel electrophoresis of cDNA amplified by the RACE method for the identification of the *nuoA* mRNA 5´ ends. The arrows point to the PCR products used to determine the mRNA 5´ ends.

The potential promoter’s similarity to sigma70 consensus, its location from 5´ end of mRNA and ability to activate the expression of a test-gene were considered. The transcription start site of the *nuoA* gene was identified using the 5´-RACE method. Two 5´ ends of the mRNA were positioned; N-I 104 bp and N-II 377 bp upstream of the *nuoA* coding sequence (Fig 3A and 3B). The consensus sequence TTGACC-N_17_-TATAC/aT of the *P*. *putida* RNA polymerase sigma factor sigma70-dependent promoters approximately 7 bp upstream from the transcriptional start point [43] was used to predict the *nuoA* promoter elements of the identified 5´ end of mRNA. A putative -10 promoter element sequence TAAAAT of the promoter P_*N-I*_ was identified exactly seven nucleotides upstream of the mapped 5´ mRNA end N-I, and a putative -35 element TTTACT was identified 17 nucleotides upstream of the -10 element (Fig 3A). The second putative -10 promoter element sequence TAGAAC of P_*N-II*_ was located three nucleotides upstream of the mapped 5´ mRNA end N-II and a GTGCGC sequence identified 17 bp upstream of the putative -10 element. Thus, the second putative promoter P_*N-II*_ had a weak similarity to the *P*. *putida* sigma70-dependent promoter consensuses.

**Fig 4:**
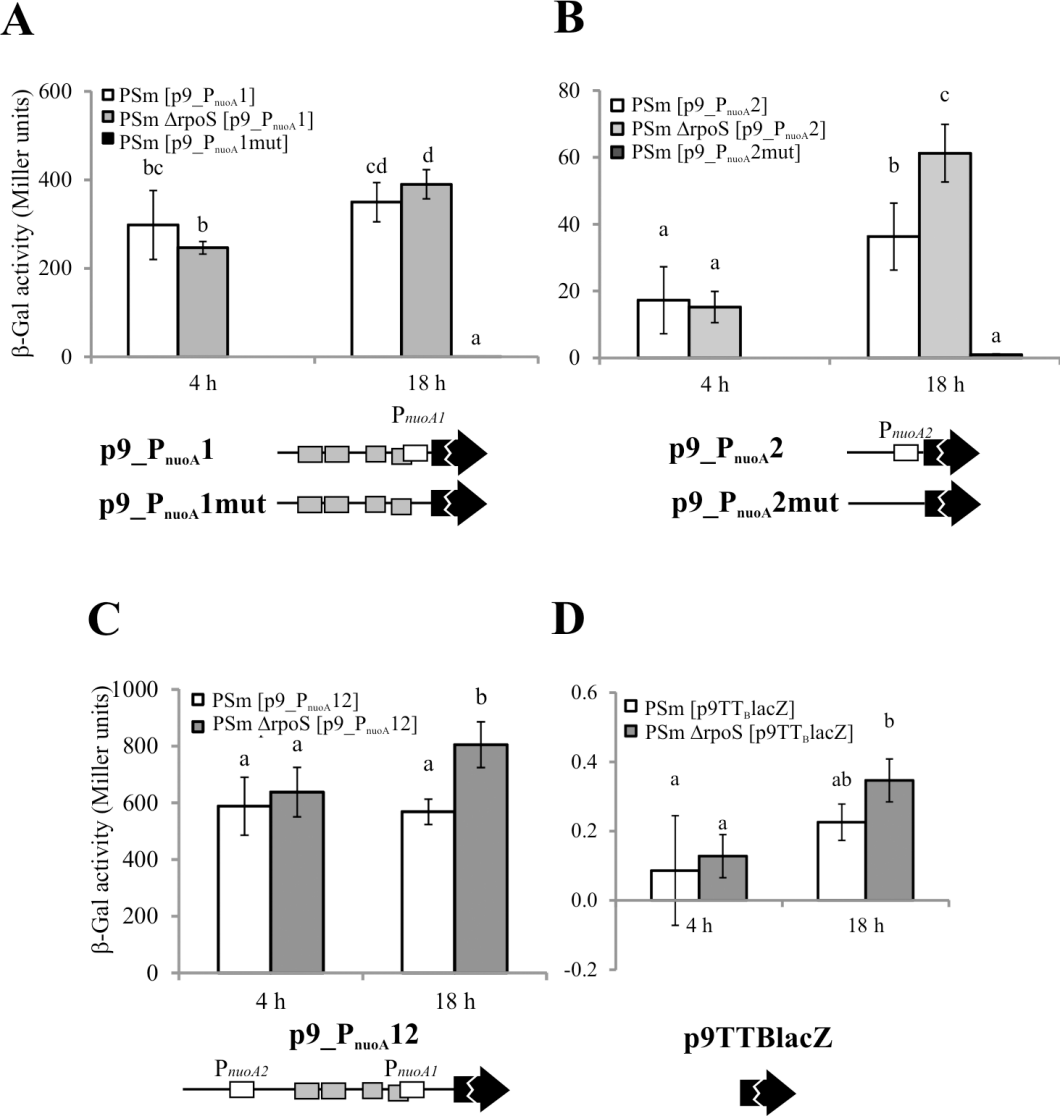
B-galactosidase activity in exponentially growing (4 h) and stationary phase (18 h) *P*. *putida* PSm and PSm ΔrpoS harboring p9TTBlacZ constructs with different length of *nuoA* promoter. Β-galactosidase (β-Gal) activity expressed from the *nuoA* promoter *lacZ* reporter constructs was measured in *P*. *putida* wild-type strain PSm and RpoS-deficient strain PSm ΔrpoS grown in LB medium 4 or 18 hours. Schemes of Fis binding sites (shown as grey boxes), promoters (shown as white boxes) are shown below the diagrams and the *lacZ* reporter gene is shown as a black arrow. Promoters are not shown in constructs with mutated -10 boxes. The scheme is not to scale. Data from at least 5 independent measurements are shown. 95% confidence intervals are shown in parentheses. Letters a-d depict different homogeneity groups according to ANOVA *post hoc* Bonferroni test. Identical letters denote non-significant differences (*P*>0.05) between averages of β-galactosidase activity.

The functionality of promoters and dependency of *nuoA-N* transcription on RpoS and Fis was assessed by β-galactosidase activity in *P*. *putida*. Therefore the promoter area of *nuoA* or part of it was cloned in front of promoterless *lacZ* gene in p9TT_B_lacZ ([29], S1 Table), which ensures low basal activity in *P*. *putida* strains [22,29]. The functionality of promoters was inspected by using p9TT_B_lacZ derivatives p9_P_nuoA_1 and p9_P_nuoA_2 that contained only one *nuoA* potential respective promoter P_*N-I*_ and P_*N-II*_ in front of the *lacZ* test gene (S1 Table, Fig 3A). The constructs p9_P_nuoA_1 and p9_P_nuoA_2 exhibited a β-galactosidase activity both in exponentially growing and stationary-phase cells of the wild-type strain PSm (Fig 4). In order to further verify the functionality of the identified promoter sequences, the A/T nucleotides were substituted with C-nucleotides in the putative -10 boxes resulting in constructs p9_P_nuoA_1mut and p9_P_nuoA_2mut (S1 Table and Fig 3A). The mutations in these putative -10 elements dramatically decreased the LacZ activity in stationary-phase cells, ensuring only a basal level of LacZ activity comparable to that measured with the negative control plasmid p9TTBlacZ (Fig 4). Thus, the substantial decline of transcription by mutations in the putative -10 elements, located at defined distances from the 5´ end of mRNA indicates the importance of these sequences for transcription initiation and the presence of functional promoters.

To investigate the possible RpoS-dependency of individual promoters, the β-galactosidase activity was compared in the *rpoS* knock-out strain PSmΔrpoS and wild-type strain PSm (Fig 4). In case of RpoS-dependent promoters the decrease of LacZ activity in PSmΔrpoS was expected in stationary-phase cells when RpoS is abundant in *P*. *putida* but not in exponentially growing bacteria, as RpoS is downregulated during fast growth of bacteria. Neither promoters were directly regulated by RpoS as the lack of RpoS did not decrease the LacZ activity in stationary-phase cells. However, the transcription initiated from the distal promoter P_*N-II*_ was elevated in the stationary-phase cells of PSmΔrpoS compared to wild-type strain PSm, indicating an indirect negative effect of RpoS (Fig 4). Additionally, unlike P_*N-I*_, the distal promoter P_*N-II*_ was approximately 2 times more active in stationary-phase wild-type cells than in exponentially growing cells (Fig 4).

In sum, the *nuoA-N* operon has two functional promoters and RpoS downregulates the transcription of the distal promoter indirectly.

### Localization of the Fis-binding sites in the *nuoA-N* promoter region

**Fig 5:**
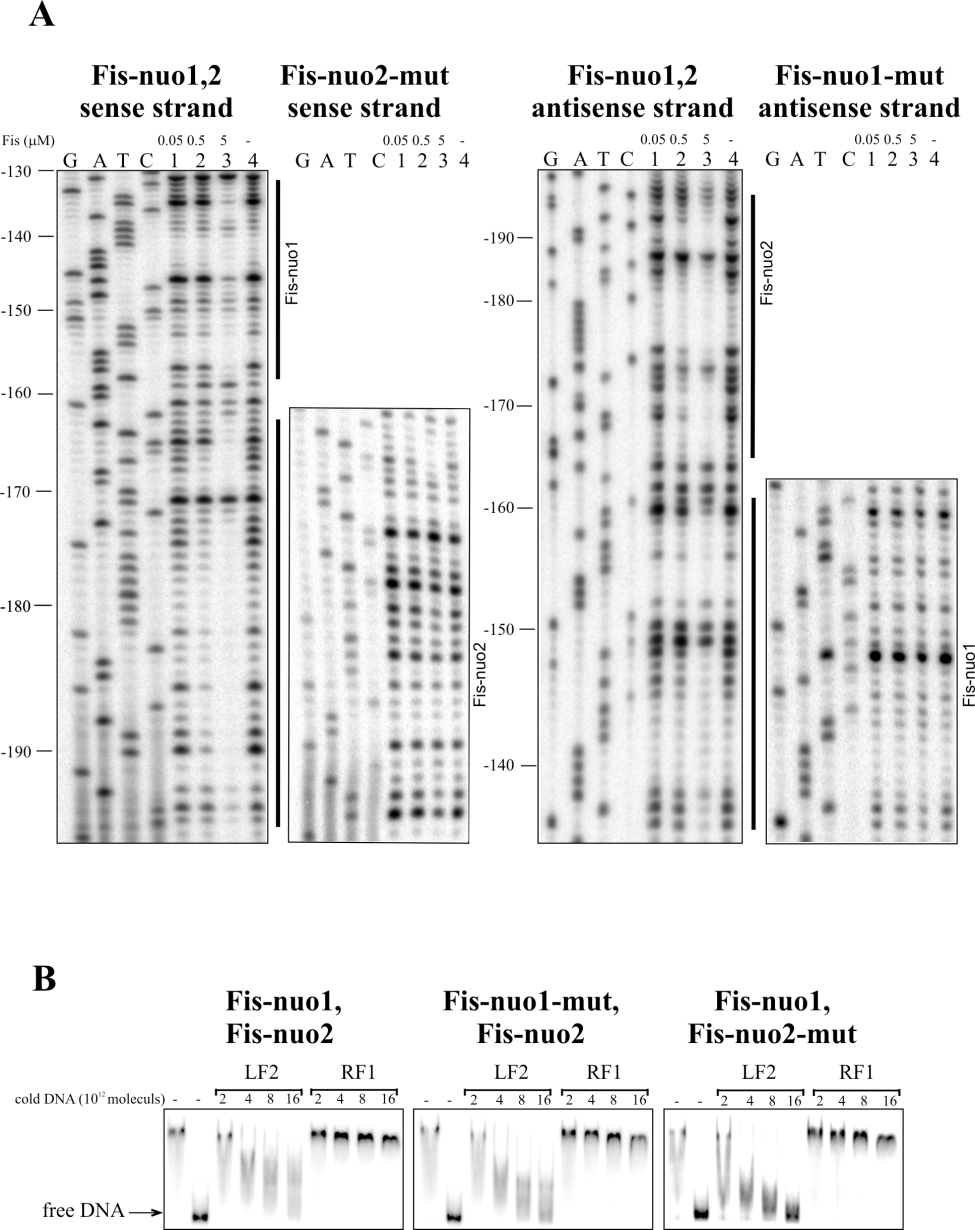
Fis binding to Fis-nuo1 and Fis-nuo2 sites upstream of the *nuoA* gene. (A) DNase I footprint analysis of the Fis binding sites Fis-nuo1 and Fis-nuo2 in the *nuoA* promoter region. Lines at the right side of the panels show the regions protected by Fis from DNase I cleavage indicating the location of Fis binding sites. (B) Gel shift assay of Fis binding to the *nuoA* promoter DNA. 2 × 10^10^ molecules of radioactively labelled PCR products containing Fis-nuo1-Fis-nuo2, Fis-nuo1-mut-Fis-nuo2, or Fis-nuo1-Fis-nuo2-mut sequences were used for Fis binding assay. Fis was outcompeted from Fis-DNA complex with unlabelled PCR product containing the Fis binding site (LF2) and PCR product without Fis-binding site (RF1). Added unlabelled DNA was calculated in molecules. 0.46 μM Fis was used in each reaction mixture except for mixtures without Fis.

**Fig 6:**
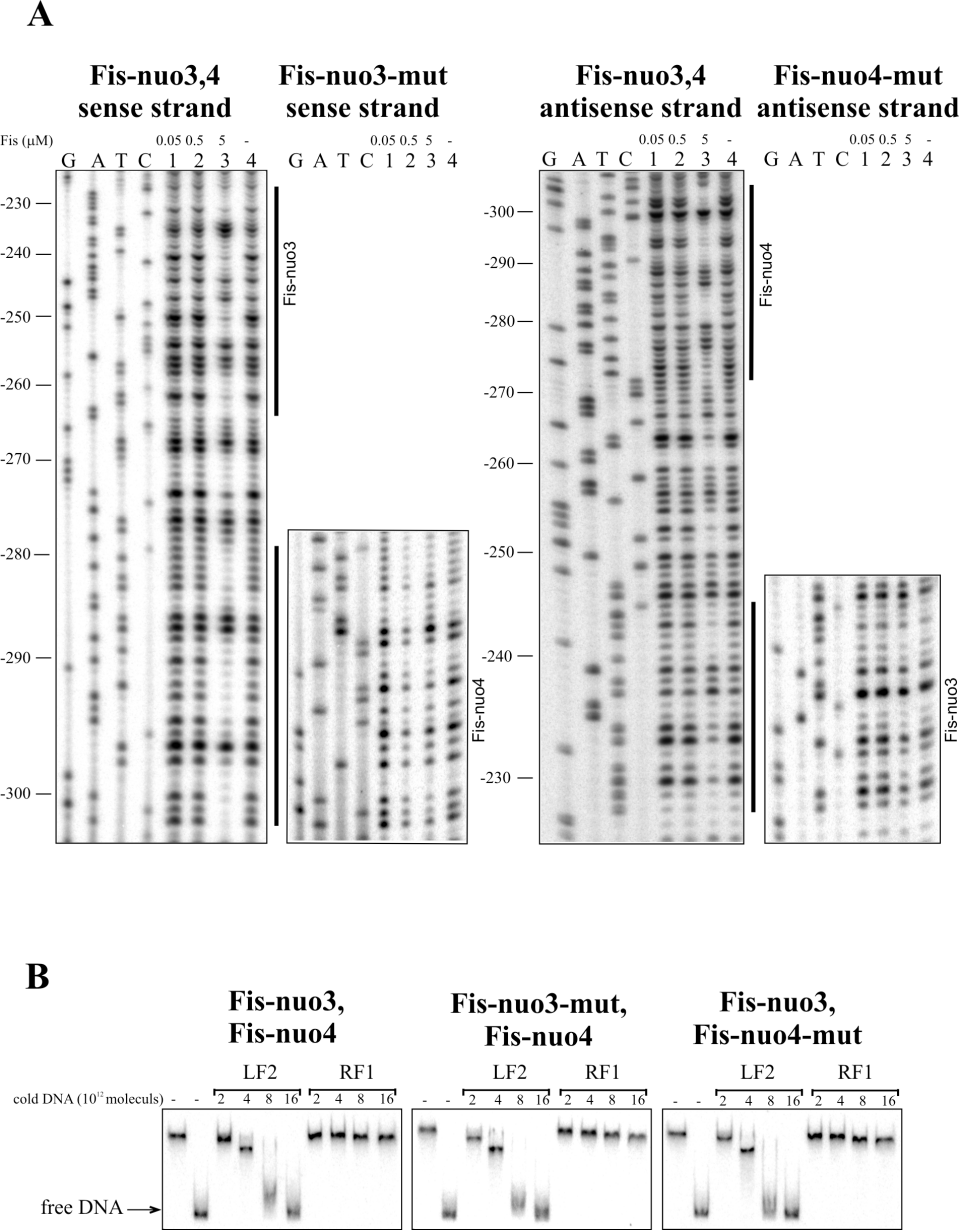
Fis binding to Fis-nuo3 and Fis-nuo4 sites upstream of the *nuoA* gene. (A) DNase I footprint analysis of the Fis binding sites Fis-nuo3 and Fis-nuo4 in the *nuoA* promoter region. Lines at the right side of the panels show the regions protected by Fis from DNase I cleavage. (B) Gel shift assay of Fis binding to the *nuoA* promoter DNA. 2 × 10^10^ molecules of radioactively labelled PCR products containing Fis-nuo3-Fis-nuo4, Fis-nuo3-mut-Fis-nuo4, or Fis-nuo3-Fis-nuo4-mut sequences were used for Fis binding. Fis was outcompeted from Fis-DNA complex with unlabelled PCR product containing the Fis binding site (LF2) and PCR product without Fis-binding site (RF1). Added unlabelled DNA was calculated in molecules. 0.46 μM Fis was used in each reaction mixture except for mixtures without Fis.

Four putative Fis-binding sites upstream of the *nuoA* gene start codon were predicted *in silico*: Fis-nuo1, Fis-nuo2, Fis-nuo3 and Fis-nuo4 (Table 1, Fig 3A). DNase I footprint analysis verified Fis binding to the four sites, which centre located approximately −145 bp (Fis-nuo1), -175 bp (Fis-nuo2), -235 bp (Fis-nuo3) and -290 bp (Fis-nuo4) upstream of the *nuoA* start codon (Figs 3A, 5A and 6A). To confirm Fis binding to the promoter region of the *nuoA* gene, we mutated five to seven nucleotides of the predicted Fis-binding sites (Fig 3A) that are described as the most critical nucleotides for Fis binding in *E*. *coli* [44]. Indeed, the DNase I footprint analysis carried out with the mutated DNA, and purified Fis revealed that the Fis binding was reduced to all of the mutated DNA sequences (Figs 5 and 6).

**Table 1:** *In silico* predicted Fis-binding sites in the upstream region of the *nuoA* gene.

Fis-binding site	Strand	Score^a^	*P*-value	Position	Sequence
Fis-nuo1	antisense	7.1	1.0×10^−4^	-145 to -164	CTGTCGCAAATTTATTCGTA
Fis-nuo2	Sense	7.4	7.0×10^−5^	-188 to -169	ACGAACGTTTTTTGTACTTA
Fis-nuo3	Sense	7.7	4.8×10^−5^	-247 to -228	AAGAAACATAATTAACAAAA
Fis-nuo4	Sense	7.6	5.4×10^−5^	-301 to -282	CAGTTCAAATGATATTAATT

^a^The applied matrix´s maximum weight score was 12.5, and minimum weight score was -28.

The binding of Fis to the upstream DNA region of the *nuoA* coding sequence was assessed by gel mobility shift analysis. We used labelled PCR-products that contained two Fis-binding sites since Fis-nuo1 and Fis-nuo2 were located close to each other and Fis-nuo3 and Fis-nuo4 were also positioned nearby (Fig 3). Unlabelled DNA containing the Fis-binding site LF2 from the left end of Tn*4652* and unspecific DNA of RF1 from the right end of Tn*4652* to determine the specificity of Fis binding to these sequences, [38] were used to outcompete Fis from the *nuoA* promoter DNA-Fis complex. The DNA of *nuoA* promoter region with the Fis binding sites can be outcompeted by the unlabelled DNA of LF2 but not with the unspecific DNA of RF1 (Figs 5B and 6B). DNA with mutated Fis binding sequence Fis-nuo2-mut (containing intact Fis-nuo1) can be outcompeted from the Fis-DNA complex by the LF2 DNA more efficiently than the wild-type DNA (Fig 5B). However, non-labelled LF2 DNA outcompeted *nuoA* promoter region with mutated Fis-nuo1-mut (containing intact Fis-nuo2) DNA less efficiently than DNA with mutated Fis-nuo2 (Fig 5B), indicating that Fis binding site Fis-nuo2 is more affine for Fis binding *in vitro*. However, the out competition of the DNA with mutated Fis binding sequence Fis-nuo3-mut or Fis-nuo4-mut (containing respectively intact Fis-nuo4 or Fis-nuo3) from the Fis-DNA complex was only slightly stronger than the out competition of the wild-type DNA by the LF2 DNA. In sum, these results confirmed that Fis could bind to the *nuo* promoter *in vitro*.

### Fis activates the transcription of *nuoA*

**Fig 7:**
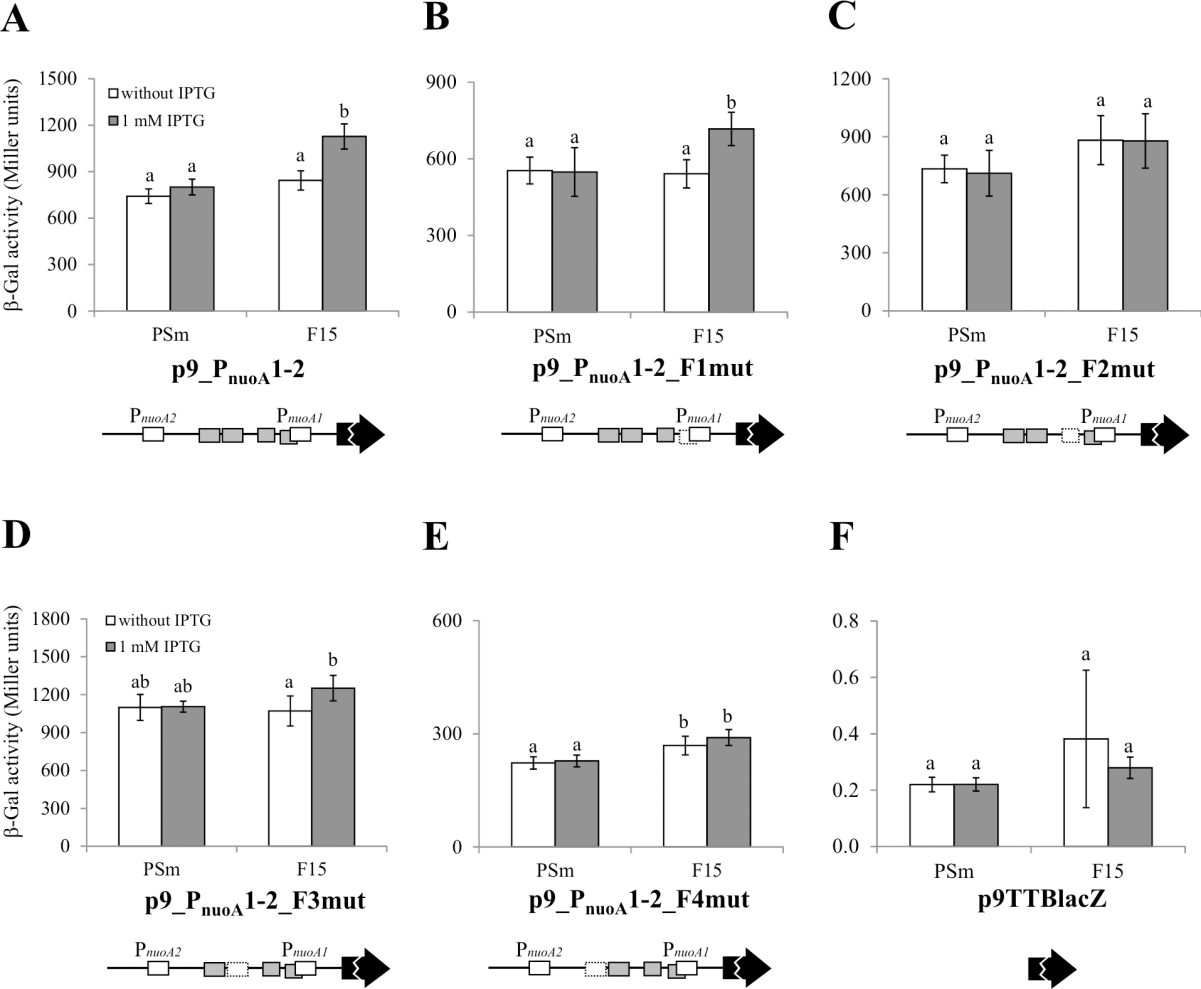
The effect of mutated Fis-binding site in *nuoA* promoter area to the level of the reporter gene *lacZ* expression in *P*. *putida*. Β-galactosidase (β-Gal) activity expressed from the *nuoA* promoter *lacZ* reporter constructs was measured in *P*. *putida* wild-type strain PSm and *fis* overexpression strain F15 grown in LB medium with or without 1 mM IPTG for 18 hours. Schemes of Fis binding sites (shown as grey boxes) are shown below the diagrams. Dotted lines denote mutated Fis binding sites and the *lacZ* reporter gene is shown as a black arrow. The scheme is not to scale. Data from at least 5 independent measurements are shown. 95% confidence intervals are shown in parentheses. Statistical analysis was carried out separately for every construct. Letters a-c depict different homogeneity groups according to ANOVA *post hoc* Bonferroni test. Identical letters denote non-significant differences (*P*>0.05) between averages of β-galactosidase activity.

The LacZ activity was assessed in *P*. *putida* wild-type strain PSm and in the *fis*-overexpression strain F15 harbouring the p9_P_nuoA_12 or its derivatives. The p9_P_nuoA_12 construct contains promoters P_*N-I*_ and P_*N-II*_, and four Fis-binding sites (S1 Table). The p9_P_nuoA_12 derivatives were obtained by mutating one out of four Fis-binding sites enabling to assess the impact of the individual Fis-binding sites on the transcription of *nuoA-N*. These point mutations in Fis binding sites also reduced Fis binding in the *in vitro* assays (Fig 3). The basal activity of LacZ in mentioned stains carrying promoterless vector p9TTBlacZ is very low, less than 0.5 Miller Units (Figs 4 and 7).

At first, the impact of IPTG on the LacZ activity in *P*. *putida* wild-type strain PSm was assessed. IPTG did not have any statistically significant effects on the β-galactosidase activity measured in PSm carrying the p9TTBlacZ derivatives (Fig 7). Thus, IPTG itself did not affect the LacZ activity but activated the expression of *fis* from the P_*tac*_ promoter in F15.

The overexpression of *fis* in F15 [p9_P_nuoA_12] increased the LacZ activity 1.3 times compared to F15 and 1.5 times compared to PSm both grown without IPTG (P < 0.001; Fig 7), indicating that Fis overexpression increases the level of the transcription of *nuoA-N* operon.

*P*. *putida* F15 carrying constructs p9_P_nuoA_12-F2mut ensured comparable LacZ activity in F15 cells either grown with or without IPTG (Fig 7). Thus, the Fis-binding site Fis-nuo2 is essential for Fis enhanced transcription from the *nuoA-N* operon promoter region (Fig 7). Mutating the Fis-binding site Fis-nuo4 reduced Fis-enhanced *nuoA-N* transcription (Fig 7), indicating to its involvement in regulation of *nuoA-N* operon transcription. Surprisingly, the mutations in the Fis-binding site Fis-nuo4 decreased the overall LacZ activity in both the PSm and F15 cells approximately 3 times (Fig 7). Mutating the other Fis binding sites (Fis-nuo1 and Fis-nuo3) influenced the overall LacZ activity no more than 1.3 times in the wild-type strain PSm and maintained the transcriptional enhancement by *fis-*overexpression (Fig 7).

In sum, this assay revealed that Fis facilitates the transcription of the *nuoA-N* operon and Fis-binding to the sites Fis-nuo2 and Fis-nuo4 is essential for the observed effects.

## Discussion

Root colonization is affected by both abiotic and biotic factors near plant roots that can have harmful or, on the contrary, beneficial effects. For successful colonization, bacteria have to recognize the presence of the plant, cope with adverse effect of detrimental factors, and successfully attach to the plant root surface.

We have previously described that *fis*-overexpression increases both biofilm formation and the expression of *lapA* in *P*. *putida* [22,23,31]. LapA is a cell surface protein necessary for *P*. *putida* attachment to biotic and abiotic surfaces [23,45]. Thus, *fis*-overexpression in *P*. *putida* should increase the attachment of bacteria to barley roots and not decline the first stages of colonization as described previously [31]. We hypothesized that Fis might be involved in the regulation of ROS-tolerance in *P*. *putida* and the decreased adherence of the *fis*-overexpression strain F15 in the first steps of colonization can be the result of increased sensitivity to exogenous ROS.

Indeed, the overexpression of *fis* in *P*. *putida* reduced the H_2_O_2_-tolerance of the cells, and as expected, adding an extra catalase gene *katA* alleviated the sensitivity of the *fis*-overexpression strain to ROS (Fig 1B). Moreover, the overexpression of *fis* in *P*. *putida* increased the amount of endogenous ROS and reduced the ability to colonize barley roots, which was decreased even more when the extracellular amount of ROS was increased by gallic acid supplementation (Fig 2). At the same time, the wild-type strain was not sensitive to the gallic acid increased ROS on barley roots (Fig 2C). We suggest that the sensitivity to ROS on barley roots may depend on the physiological conditions of the bacterial cells. Most probably, the wild-type strain was able to use a compensatory mechanism to detoxify the exogenous ROS emitting from barley roots, and *P*. *putida* with the overexpressed *fis* was not. This raised the question whether Fis could influence ROS-tolerance by regulating the transcription of some specific genes.

We performed transposon mutagenesis to identify Fis-dependent genes that may regulate ROS tolerance of bacteria and identified 19 mini-Tn*5* insertions into the F15 *nuoA-N* operon genes. This finding indicates that the regulation of the *nuoA-N* operon by Fis could be involved in the ROS-tolerance as an alternative mechanism to reduce endogenous ROS. Indeed, the deletion of *nuoA-N* decreased endogenous ROS in the *fis*-overexpressing cells (Fig 2B) and increased the tolerance to exogenous ROS (Fig 1B). The genes *nuoA* to *nuoN* encode NuoA-N proteins that assemble to NADH dehydrogenase I in the cytoplasmic membrane [15,46,47]. This complex is a respiratory-chain enzyme that catalyses the transfer of two electrons from NADH to quinones in a reaction that is associated with proton translocation across the membrane [48].

Two hypotheses could explain how NADH dehydrogenase I may affect the amount of endogenous ROS: generating ROS itself or depleting reductive agent NADH. It has been shown that during electron transport the NADH dehydrogenase I may produce ROS in *Escherichia coli* [16]. In this case, ROS may arise during NADH oxidation and electron transport through the cofactor FMN [16]. Alternatively, it is possible that depletion of NADH, as a reducing agent, can increase the endogenous ROS. For example, the presence of antibiotics in the growth medium induces many oxidative stress genes, e.g. NADH peroxidase and glutathione reductase [21], and thereby bacteria need NADH to use it for ROS detoxification. Thus, the forced expression of *nuoA-N* genes by *fis*-overexpression may cause a deficit in NADH, disabling the detoxification of endogenous ROS. Whichever hypothesis is true the sum of endogenous and exogenous ROS for F15ΔnuoA-N is lower resulting in higher tolerance of bacteria to exogenous ROS and increased colonization efficiency (Figs 1B, 2C and 2D). Moreover, unlike F15, the colonization efficiency of F15ΔnuoA-N was improved in the *fis*-overexpression background (Fig 2C). It seems that the attachment of *P*. *putida* depends on two factors: detoxification of ROS and the presence of the main adhesin LapA as we have previously reported that *fis*-overexpression increases the expression of *lapA* [22,23]. Thus, the elevated amount of LapA on cells surface would enhance attachment to roots if the bacteria were capable to effectively detoxify ROS.

The identification of mini-Tn*5* insertions in several other genes responsible for the maintenance of the reductive force support the NAD(P)H depletion hypothesis. Although only a few mini-Tn*5* insertions into the methionine biosynthesis genes were selected, the mini-Tn*5* insertion in the PP_5275 and *metR-1* (PP_1063) restored the H_2_O_2_-tolerance to the wild-type level despite of *fis*-overexpression (S3 Table). It is known that ROS generate thiol stress by oxidizing thiol residues in biomolecules, especially in proteins, creating disulphide bonds that can inactivate enzymes [49]. Therefore, bacteria need reductive force to reduce disulphide bonds in proteins and NADPH used in methionine biosynthesis is a powerful reductive agent for detoxification of ROS in bacteria [50,51]. Thus, the increased amount of Fis may affect the reduction of oxidized biomolecules in *P*. *putida* as it keeps up the methionine biosynthesis that would be down-regulated in natural circumstances. Because plant roots secrete amino acids to attract plant growth promoting bacteria [52,53], it is more likely that amino acids biosynthesis is downregulated in bacteria near plant roots. The reason of downregulation is probably not only to conserve energy but also to avoid the depletion of reductive agents like NADPH.

Additionally, ketoacids can neutralize ROS in an NADPH-independent manner [54]. For example, the excess of ROS enhances accumulation of alpha-ketoglutarate, the Krebs cycle metabolite that is used for reducing disulphide bonds in *Pseudomonas fluorescens* [54]. Therefore, it is possible that the insertion of mini-Tn*5* in the genes of methionine biosynthesis and alpha-ketoglutarate metabolism genes alleviates thiol stress in the *fis*-overexpression background (S3 Table). Thus, during the colonization process bacteria could regulate their metabolism to cope with the toxic effect of exogenous ROS by downregulating the expression of the *nuoA-N* operon and increasing the reductive force by keeping the amount of NADH, NADPH and ketoacids high.

As a significant number of transposon mutants had transposon insertions in the *nuo* operon genes and four potential Fis-binding sites were predicted in the upstream sequence of the *nuoA* gene, we studied the regulation of this operon by Fis in depth. The organization of the *nuoA-N* genes is similar in different bacteria: they are located in one operon and are co-transcribed in *E*. *coli*, *Salmonella enterica* and *P*. *fluorescens* [15,55,56]. The presence of the similar *nuo* operon structure in *P*. *putida* implies that the *nuoA-N* operon genes could be co-transcribed in *P*. *putida* as well.

The *nuoA-N* operon in *P*. *putida* has two promoters in front of the *nuoA* gene and these promoters are down regulated indirectly by RpoS (Fig 4). Fis binds to multiple binding sites upstream of the Fis-dependent promoter (Figs 5 and 6) and upregulates the transcription (Fig 7). These results indicate that transcription of *nuo*-operon in *P*. *putida* is enhanced in the presence of nutrients, when bacteria have energy to neutralize ROS; and down regulated by stress, when availability of detoxification of additional ROS is decreasing. Our results revealed that two Fis-binding sites affect the transcription of the *P*. *putida nuoA* positively. The proximal Fis binding site Fis-nuo2 is located approximately -70 bp from the 5´ end of the *nuoA* mRNA N-I (Fig 3). Such distance is characteristic to activators that directly interact with the RNA polymerase [57]. The region, indicated as Fis-nuo4, seems to be involved in the transcriptional regulation as well, probably through the modulation of DNA topology. The Fis-nuo4 is located -290 bp from the 5´ end of the *nuoA* mRNA N-I (Fig 3) and can activate transcription from this distance only by generating a DNA loop. Therefore we propose that binding of Fis to Fis-nuo4 could change the DNA topology that enhances the transcription of the *nuoA-N* operon.

To conclude, in this study we have described the involvement of the global regulator Fis in ROS tolerance and barley root colonization of *P*. *putida*. The negative effect of Fis on the ROS-tolerance and thereby on the root colonization efficiency could appear via enhancement of the transcriptional level of the *nuoA-N* operon and the accumulation of endogenous ROS. Fis binds to the promoter region of the *nuoA-N* operon and facilitates its transcription by binding to Fis-nuo2 and Fis-nuo4 sites upstream of the operon promoter.

## Supporting information

S1 Table Bacterial strains and plasmids used in this study. (PDF)

S2 Table Oligonucleotides used in this study. (PDF)

S3 Table P. putida KT2440 genes for which disruption with mini-Tn5 increased NQO and ROS-tolerance of F15 cells in Fis-overexpression conditions. (XLSX)
