# Supplementary material for: Colonization efficiency of Pseudomonas putida is influenced by Fis-controlled transcription of nuoA-N operon
Source: PLoS One. 2018 Aug 2;13(8):e0201841. doi: 10.1371/journal.pone.0201841 (PMC6072106; doi:10.1371/journal.pone.0201841)
Supplement: S1 Table — (PDF) [file pone.0201841.s001.pdf]

S1 Table. Bacterial strains and plasmids used in this study

**Strains and plasmids**	**Genotype or description**	**Source/reference**
E. coli		
DH5αλ*pir*	λ*pir* lysogen of DH5α	(1)
HB101[pRK2013]	Helper strain for conjugation containing pRK2013 plasmid	(2)
P. putida		
PaW85	Isogenic to KT2440	(3)
PSm	PaW85; chromosomal mini-Tn*7*-ΩSm1 (Sm^r)	(4)
PSm ΔrpoS	PSm; Δ*rpoS* (Sm^r)	(5)
F15	PaW85; chromosomal mini-Tn*7*-ΩGm-term-*lacI*^{q}-P$_{tac}$-*fis*-T1T2 (Gm^r)	(4)
F15 ΔnuoA-N	F15; ΔPP4119- ΔPP4131 (Gm^r)	This study
Plasmids		
p9TTB	pPR9TT derivative without *lacZ* gene (Cm^r, Amp^r)	(6)
p9TTBlacZ	Promoter probe pPR9TT derivative containing full-length *lacZ* from pKRZ-1(Cm^r , Amp^r)	(6)
p9_katA	p9TTB carrying catalase gene *katA*	This study

pKStackatA	Vector containing the P_{tac}-*katA* transcriptional fusion	(7)
pBAM1	Suicide vector carrying mini-Tn*5* with kanamycin resistance gene (Km^r, Amp^r)	(8)
pEMG	Suicide vector for knockout mutagenesis (Km^r)	(1)
pEMG-ΔnuoA-nuoN	Suicide vector pEMG with 351-bp of upstream and 534-bp of downstream DNA of the *nuoA-N* operon inserted between the *Bam*HI sites (Km^r)	This study
pSW (Sce-I)	I-SceI-expressing plasmid (Amp^r)	(9)
pLA1-12	Carrying LF2 site in the left end DNA of Tn*4652*; (Amp^r)	(10)
pRA1-12	Carrying RF1 site in the right end DNA of Tn*4652*; (Amp^r)	(10)
p9_P_{nuoA}1	The 249-bp-long promoter region of the *nuoA* gene containing potential promoter $P_{N\text{-}I}$ cloned into p9TTBlacZ BamHI site (Cm^r, Amp^r)	This study
p9_P_{nuoA}2	The 191-bp-long promoter region of the *nuoA* gene containing potential promoter $P_{N\text{-}II}$ cloned into p9TTBlacZ BamHI site (Cm^r, Amp^r)	This study
p9_P_{nuoA}1mut	The 249-bp-long promoter region of the *nuoA* gene containing 4 substitutions in potential -10 box of promoter $P_{N\text{-}I}$ cloned into p9TTBlacZ	This study

	BamHI site (Cm^r, Amp^r)	
p9_P_{nuoA}2mut	The 191-bp-long promoter region of the *nuoA* gene containing 4 substitutions in potential -10 box of promoter $P_{N\text{-}II}$ cloned into p9TTBlacZ BamHI site (Cm^r, Amp^r)	This study
p9_P_{nuoA}12	The 567-bp-long promoter region of the *nuoA* gene containing potential promoters $P_{N\text{-}I}$ and $P_{N\text{-}II}$ cloned into p9TTBlacZ BamHI site (Cm^r, Amp^r)	This study
p9_P_{nuoA}12-F1mut	The 567-bp-long promoter region of the *nuoA* gene with mutated Fis-nuo1 site cloned into p9TTBlacZ BamHI site (Cm^r, Amp^r)	This study
p9_P_{nuoA}12-F2mut	The 567-bp-long promoter region of the *nuoA* gene with mutated Fis-nuo2 site cloned into p9TTBlacZ BamHI site (Cm^r, Amp^r)	This study
p9_P_{nuoA}12-F3mut	The 567-bp-long promoter region of the *nuoA* gene with mutated Fis-nuo3 site cloned into p9TTBlacZ BamHI site (Cm^r, Amp^r)	This study
p9_P_{nuoA}12-F4mut	The 567-bp-long promoter region of the *nuoA* gene with mutated Fis-nuo4 site cloned into p9TTBlacZ BamHI site (Cm^r, Amp^r)	This study

1. Martinez-Garcia E, de Lorenzo V. Engineering multiple genomic deletions in Gram-negative bacteria: analysis of the multi-resistant antibiotic profile of *Pseudomonas putida* KT2440. Environmental microbiology. 2011;13(10):2702-16.

2. Boyer HW, Roulland-Dussoix D. A complementation analysis of the restriction and modification of DNA in *Escherichia coli*. Journal of molecular biology. 1969;41(3):459-72.

3. Bayley SA, Duggleby CJ, Worsey MJ, Williams PA, Hardy KG, Broda P. Two modes of loss of the Tol function from *Pseudomonas putida* mt-2. Mol Gen Genet. 1977;154(2):203-4.

4. Jakovleva J, Teppo A, Velts A, Saumaa S, Moor H, Kivisaar M, et al. Fis regulates the competitiveness of *Pseudomonas putida* on barley roots by inducing biofilm formation. Microbiology (Reading, England). 2012;158(Pt 3):708-20.

5. Ainelo H, Lahesaare A, Teppo A, Kivisaar M, Teras R. The promoter region of lapA and its transcriptional regulation by Fis in Pseudomonas putida. PloS one. 2017;12(9):e0185482. Epub 2017/09/26.

6. Kivistik PA, Putrins M, Puvi K, Ilves H, Kivisaar M, Hõrak R. The ColRS two-component system regulates membrane functions and protects *Pseudomonas putida* against phenol. Journal of bacteriology. 2006;188(23):8109-17.

7. Tarassova K, Tegova R, Tover A, Teras R, Tark M, Saumaa S, et al. Elevated mutation frequency in surviving populations of carbon-starved *rpoS*-deficient *Pseudomonas putida* is caused by reduced expression of superoxide dismutase and catalase. Journal of bacteriology. 2009;191(11):3604-14. Epub 2009/04/07.

8. Martinez-Garcia E, Calles B, Arevalo-Rodriguez M, de Lorenzo V. pBAM1: an all-synthetic genetic tool for analysis and construction of complex bacterial phenotypes. BMC microbiology. 2011;11:38. Epub 2011/02/24.

9. Wong SM, Mekalanos JJ. Genetic footprinting with mariner-based transposition in *Pseudomonas aeruginosa*. Proceedings of the National Academy of Sciences of the United States of America. 2000;97(18):10191-6. Epub 2000/08/30.

10. Teras R, Hõrak R, Kivisaar M. Transcription from fusion promoters generated during transposition of transposon Tn*4652* is positively affected by integration host factor in *Pseudomonas putida*. Journal of bacteriology. 2000;182(3):589-98. Epub 2000/01/14.
