# Supplementary material for: Colonization efficiency of Pseudomonas putida is influenced by Fis-controlled transcription of nuoA-N operon
Source: PLoS One. 2018 Aug 2;13(8):e0201841. doi: 10.1371/journal.pone.0201841 (PMC6072106; doi:10.1371/journal.pone.0201841)
Supplement: S2 Table — (PDF) [file pone.0201841.s002.pdf]

S2 Table Oligonucleotides used in this study

Oligonucleotide designation [a]	Oligonucleotide sequence [b]	Complementary region
Adapt-lyh	5´-GACTCGAGTCGACATCG-3´	Adaptor primer that complements the Adapt_pikkC stretch and adds a special adaptor sequence to the 5´ end of each cDNA
Adapt_pikkC	5´-GACTCGAGTCGACATCGA$(C)_{17}$-3´	Poly (C) - tail
ARB2	5´-GGCACGCGTCGACTAGTAC-3´	Complementary to the sequence of PCR products obtained in the first round of arbitrary PCR
ARB6	5´-GGCACGCGTCGACTAGTAC$(N)_{10}$ACGCC-3´	Arbitrary primer; *P. putida* genome contains 12050 instances of the ACGCC linker sequence
fis-BamHI (BamHI)	5´-AGA<u>GGATCC</u>TTACAACAAGTCGTACTGC-3´	Positions 321 to 303 from the start codon of the *fis* gene (PP4821)
ME-I-uus	5´-GCTGAATATGGCTCATAACACCC-3´	Positions 139 to 120

		from the IE end of mini-Tn*5* in the pBAM1
ME-I-uus2	5´-TATCTTGTGCAATGTAACATCAGAG-3´	Positions 70 to 56 from the IE end of mini-Tn*5* in the pBAM1
nuoA-RACE2	5´-GTTGTGGGCGATGAGTCC-3`	Positions 33 to 16 from the start codon of the *nuoA* gene (PP4119)
nuoA-RACE3	5´-AGCATCGCGACCAGATAG-3´	Positions 215 to 198 from the start codon of the *nuoA* gene (PP4119)
nuo1-mut	5´-ATCCTACGAATAA**GG**TTGCGA**GTCG**AATTTTACTTGCAC-3´	Positions -168 to -130 from the start codon of the *nuoA* gene (PP4119)
nuo2-mut	5´-GTTACGAACGTTT**G**T**CC**TA**CC**TAATCCTACGAATAA-3´	Positions -191 to -156 from the start codon of the *nuoA* gene (PP4119)
nuo3-mut	5´-GCATTACGCGCGGTT**GGC**T**G**A**C**TTATGTTTCTTGCAG-3´	Positions -215 to -252 from the start codon of the *nuoA* gene (PP4119)
nuo4-mut	5´-GTAATGATAATTAA**GG**TC**GG**T**GCC**ACTGCTGCGCTCGAA-3´	Positions -275 to -313 from the start codon of the *nuoA* gene (PP4119)

nuoAup1	5′-GCGCGTAATGCCCGAATT-3′	Positions -225 to -207 from the start codon of the *nuoA* gene (PP4119)
nuoAdown1	5′-CGTTCGTAACTGGCTGAG-3′	Positions -182 to -199 from the start codon of the *nuoA* gene (PP4119)
nuoAdown2	5′-CAGTGACCAAATGTCGCAG-3′	Positions -76 to -94 from the start codon of the *nuoA* gene (PP4119)
nuo-1-fw (BamHI)	5′-TGGATCCTGTTCGTGTTCTCCGA-3′	Positions -567 to -550 from the start codon of the *nuoA* gene (PP4119)
nuo-1-rev	5′-GCTAGAAATCCTTATACAGA-3′	Positions -1 to -19 from the start codon of the *nuoA* gene (PP4119)
nuo-2-rev (BamHI)	5′-TGGATCCGGCTTAAACTACTCGTTATG-3′	Positions 1967 to 1948 from the start codon of the *nuoN* gene (PP4131)
nuo-2-fw	5′-CTGTATAAGGATTTCTAGCGATGGTTCAGCAAGCGGG-3′	Positions 1434 to 1451 from the start codon of the *nuoN* gene (PP4131); 19 nucleotides at the beginning of the

		oligonucleotide is complementary to the nuo-1-rev primer
PP4119-fw (BamHI)	5′-AGGATCCAATCGCCAGATGAACTTTAC-3′	Positions -351 to -332 from the start codon of the *nuoA* gene (PP4119)
PP4119-rev (BamHI)	5′-AGGATCCGCTAGAAATCCTTATACAGA-3′	Positions -1 to -19 from the start codon of the *nuoA* gene (PP4119)
PP4119-3-rev (BamHI)	5′-AGGATCCTTGTTCTAGCAG-3′	Positions -377 to -387 from the *nuoA* start codon
PP4119-3-revmut (BamHI)	5’-AGGATCCTTG**GG**C**GG**GCAGCTCTCGGTCCTGC-3’	Positions -377 to -403 from the *nuoA* start codon
pp4119-4-rev (BamHI)	AGGATCCTCGCGCTGATTTTATGGGTA-3’	Positions -104 to -123 from the *nuoA* start codon
pp4119-4-revmut (BamHI)	AGGATCCTCGCGCTG**G**TT**GGG**TGGGTA-3’	Positions -104 to -123 from the *nuoA* start codon
PRH8	5′-GCTGAGCTCAGACGGTGGATGACCAGC-3′	Positions 102 to 85 inside the right end of

		Tn*4652*
Prtac	5´-AATTAATCATCGGCTCGTATAA-3´	Positions -100 to -79 from the start codon of the *fis* gene in the mini Tn*7*-term-*lacI*^{q}-P*tac*-*fis*-T1T2-ΩGm cassette
SIDD-2	5´-AGAGCTCCTGTACGTGCGCTT-3	Positions 244 to 228 inside the left end of Tn*4652*
TnLsisse	5´-GCAAAGACTGCTTCGCGCCC-3´	positions 85 to 105 inside the left end of Tn*4652*
Tnots	5´- CGTGGGGTTATGCCGAGATAAGGC- 3´	Positions 10 to 19 upstream from the left end of transposon Tn*4652*

[a] Restrictases are shown in brackets

[b] Restriction sites are underlined, substituted nucleotides are shown bold
